# High-magnification inpatient cases under DIP payment reform: prevalence, cost burden, and risk factors in an NHC-administered hospital in China

**DOI:** 10.3389/fpubh.2026.1915516

**Published:** 2026-09-03

**Authors:** Jianjun Lu, Zhuochen Lin, Ziyi Xin, Junfeng Liu, Yuting Gu, Ying Xiong, Wujun Zhang

**Affiliations:** Department of Quality Management and Evaluation, The First Affiliated Hospital, Sun Yat-Sen University, Guangzhou, China

**Keywords:** diagnosis-intervention packet, high-magnification cases, hospitalization costs, influencing factors, tertiary hospitals

## Abstract

**Background:**

The nationwide rollout of Diagnosis-Intervention Packet (DIP) payment reform in China aims to enhance inpatient cost efficiency, yet high-magnification cases impose escalating financial burdens on tertiary hospitals. This study characterized the cost profiles and independent determinants of high-magnification cases under DIP to inform precision cost governance and medical insurance policy optimization.

**Methods:**

This retrospective observational study included 191,490 consecutive inpatient admissions from a National Health Commission-administered hospital in southern China (2024–2025). High-magnification cases were defined as actual hospitalization costs exceeding 2 times the standard DIP reimbursement benchmark in the present study. We analyzed their clinical features, cost composition, and multi-dimensional distribution. Multi-variable logistic regression adjusted for department-level fixed effects was applied to identify independent determinants.

**Results:**

A total of 17,929 (9.36%) high-magnification cases were identified. These cases had an average hospitalization cost of 65,366.0 Chinese Yuan (CNY), which was 2.38 times the overall average of 27,444.7 CNY, and a mean length of stay of 9.1 days. 49.42% of high-magnification cases resulted in DIP payment deficits, with a mean deficit of 1,492.1 CNY per case. The Charlson Comorbidity Index (CCI) fluctuated between 1.8 and 2.0 during the study period. Using multivariate logistic regression, the confidence intervals (CI) and odds ratios (OR) of influencing factors were calculated. Independent positive predictors included length of stay (LOS) (OR = 1.139, 95% CI 1.136–1.143), CCI (OR = 1.068, 95% CI 1.057–1.080), consumable cost proportion (OR = 1.029, 95% CI 1.026–1.031), and medication cost proportion (OR = 1.053, 95% CI 1.051–1.055). Subgroup analyses further suggested the highest high-magnification risk (all *p* < 0.001) for *Secondary comprehensive disease category* (OR = 9.474, 95% CI 8.028–11.180), *Pregnancy, childbirth & puerperium* (OR = 8.162, 95% CI 4.503–14.793), *Health status & healthcare contact factors* (OR = 7.292, 95% CI 5.575–9.537) and *Skin & subcutaneous tissue disorders* (OR = 3.695, 95% CI 2.715–5.029).

**Conclusion:**

High-magnification DIP cases feature excessive resource consumption and high deficit rates. Prolonged LOS, consumables and medication costs are core drivers. Targeted cost control and optimized reimbursement benchmarks for complex conditions is beneficial for improving the utilization efficiency of medical insurance funds and maintaining the stable operation of hospitals while ensuring medical quality.

## Introduction

Escalating healthcare expenditure has posed a persistent threat to the sustainability of health systems globally, catalyzing worldwide shifts from volume-based fee-for-service payment to value-based alternative payment models (1). Diagnosis-Related Groups (DRGs), as a well-established prospective payment system, have been widely implemented across high-income economies to constrain excessive cost growth while safeguarding healthcare quality (2, 3). In China, the big data-driven Diagnosis-Intervention Packet (DIP) payment system, a domestically tailored prospective payment model that assigns standardized reimbursement scores to inpatient cases based on combinations of diagnoses and interventions, has been scaled up nationwide since 2020 as a core component of national medical insurance reform (4). As one of the earliest pilot cities, Guangzhou has fully operationalized DIP payment across its tertiary hospitals for more than seven years, offering a mature real-world context to examine the operational implications of this payment model for large medical institutions (5).

Under the DIP framework, inpatient cases are reimbursed according to pre-specified standard scores matched to their respective disease groups, which creates financial incentives for hospitals to streamline resource utilization and improve cost efficiency (6). Nevertheless, high-magnification cases, defined as cases whose actual total hospitalization cost exceeds 2 times the standard reimbursement benchmark of their assigned DIP group in Guangzhou, have emerged as a pronounced challenge for both healthcare providers and insurance authorities. On one hand, these cases subject hospitals to substantial financial deficits due to the mismatch between actual care costs and standardized reimbursement (7); on the other hand, they contribute to upward pressure on the overall expenditure of medical insurance funds (5). Existing research on DIP payment has largely centered on macro-level policy evaluation, aggregate cost-control outcomes, and comparative performance between DIP and DRG models (8). Few studies have systematically delineated the distributional patterns of high-magnification cases across clinical departments, diagnostic categories, and hierarchical DIP groups, especially in high-volume tertiary care centers that care for a disproportionately large share of complex and severe diseases (9). Additionally, the temporal trajectories of hospitalization costs and high-magnification case burden in the post-pandemic era remain under-investigated, hindering the development of targeted cost-containment strategies by hospital administrators and policymakers (10).

To address these evidence gaps, we conducted this retrospective cohort study in a National Health Commission-administered hospital in Guangzhou, where the DIP payment system has been fully operational since 2018 (4). Using comprehensive clinical and administrative data from consecutive inpatients between 2024 and 2025, this study aimed to systematically characterize the baseline profiles of inpatients under the DIP payment scheme, comprehensively analyze the distribution of high-magnification cases, hospitalization cost structure, and average length of stay across clinical departments, International Classification of Diseases−10th Revision (ICD-10) based all discharge diagnoses, and hierarchical DIP classification tiers, and further examine the quarterly temporal trends of average total hospitalization costs and high-magnification case volume over the study period. The findings of this study will help identify high-burden subgroups with elevated risk of cost overruns, and provide empirical evidence for refined hospital-level cost management, optimized resource allocation, and continuous improvement of DIP payment policies in tertiary care settings.

## Methods

### Data source

This retrospective cohort study was conducted at the First Affiliated Hospital of Sun Yat-sen University in Guangzhou, Guangdong Province, China. As an NHC-administered hospital, it serves as a regional hub for complex and critical care, with approximately 3,500 inpatient beds and over 130,000 annual inpatient discharges.

The big data-driven DIP payment system, which was initially piloted and progressively rolled out across Guangzhou, has been fully implemented in the hospital since January 1, 2018. Study data were extracted from routinely collected inpatient clinical and administrative records covering the period from January 1, 2024, to December 31, 2025.

### Study design

All consecutive inpatient admissions between January 1, 2024, and December 31, 2025, were screened for eligibility. Relevant data were extracted from two hospital information systems: the electronic medical record system of inpatients statistics and the medical insurance settlement system for medical cost data. Two independent researchers performed data extraction and cross-validation to ensure data integrity, accuracy and consistency. For subsequent analyses, admission dates were aggregated into calendar quarters to assess temporal trends. Principal diagnoses were further categorized into systemic disease groups based on ICD-10 chapter classifications to support stratified burden analysis.

#### Inclusion criteria

(1) Cases with completed inpatient treatment and final settlement under the DIP payment scheme. (2) Complete and valid data available from hospital discharge abstracts and the municipal medical insurance settlement system.

#### Exclusion criteria

(1) Cases with missing diagnosis codes or surgical procedure codes. (2) Cases with invalid total hospitalization costs (≤ 0 Chinese Yuan, CNY); (3) Cases involving inter-hospital transfer or voluntary discharge against medical advice within 24 h of admission.

#### Collected variables

(1) Demographic characteristics included age at admission, sex. (2) Clinical characteristics included discharge department, all discharge diagnoses coded per ICD-10, surgical procedures coded per the International Classification of Diseases-9th Revision, Clinical Modification (ICD-9-CM), and length of stay (LOS). (3) Cost-related variables included total hospitalization cost, medication cost, medical consumable cost, and diagnostic examination and laboratory costs. (4) Payment administrative variables included discharge date, DIP category, standard DIP score, and actual reimbursed settlement amount.

#### Variable definitions

Key variables were defined in strict accordance with Guangzhou municipal DIP payment policies and established health services research conventions: (1) The score ratio for each case refers to the ratio between the actual DIP score obtained by an individual during hospitalization and the benchmark score of the corresponding DIP group at the same hospital level. According to the official standards of high score ratios in the DIP reimbursement policy of Guangzhou, this study defined cases with a score ratio greater than 2 as high-magnification cases. This binary variable was coded as 1 in high-magnification cases and 0 in non-high-magnification cases. (2) DIP classification tiers. Six hierarchical DIP grouping categories per local grouping rules: *Tertiary core disease groups, Secondary core disease groups, Secondary comprehensive disease groups, Primary core disease groups, Primary comprehensive disease groups*, and *Multi-diagnosis disease groups*. (3) The Charlson Comorbidity Index (CCI) was used to quantitatively assess the overall burden of comorbidities for each patient. CCI is currently a widely used tool for assessing comorbidities and includes 17 types of comorbid diseases, each assigned a weight based on its impact on the risk of death. In this study, the weighted total score of CCI for each patient was calculated. A higher score indicates a greater burden of comorbidities, and this score was included as an important covariate in the multivariate logistic regression model to adjust for the differences in baseline clinical complexity among patients and to mitigate the impact of these differences on the risk of high-magnification cases. (4) Cost proportions. Medication cost proportion (MCP) was calculated as (medication cost/total hospitalization cost) × 100%; consumable cost proportion (CCP) was calculated as (medical consumable cost/total hospitalization cost) × 100%. (5) DIP payment balance. Calculated as actual total hospitalization cost minus the final DIP reimbursed amount. A positive value indicates a financial deficit for the hospital, while a negative value indicates a payment surplus. (6) Discharge quarter: Categorized into 8 consecutive calendar quarters from 2024 Q1 to 2025 Q4 for temporal trend analysis. (7) Principal diagnosis categories: Systemic disease groups classified based on ICD-10 chapter structures, including diseases of the circulatory system, diseases of the respiratory system, diseases of the digestive system, and other diagnostic categories.

Moreover, sensitivity analysis was conducted to test the robustness of the conclusion. According to the official definition standards of DIP payment policy in Guangzhou City, this study defines DIP high-amplification cases as those with actual DIP scores exceeding twice the corresponding standard DIP scores. In this study, the median cost ratio of included cases is 0.98 (interquartile range: 0.87–1.15), and the 90th, 95th, and 97.5th percentiles are 1.96, 3.12, and 4.86, respectively. The 2.0-fold threshold approximately corresponds to the 90th–95th percentiles. We defined high-cost cases using five sets of critical cost ratios: 2.0, 2.5, 3.0, 3.1, and 4.8. We repeated the fitting of the multivariate Logistic regression model and compared whether the effect direction, effect size, and statistical significance of the independent variables were consistent under different thresholds.

## Statistical analysis

The multicollinearity of the regression model was maintaining the stable operation of hospitalsriance inflation factor (VIF). Considering the differences in degrees of freedom among categorical variables, we calculated the generalized VIF and corrected it by the degree of freedom (GVIF^(1/(2 × df))), and set the corrected value < 2.0 as the criterion for acceptable multicollinearity. To distinguish the independent effects of patient inherent baseline factors and treatment process-related factors on high-dose cases, a stratified multivariate Logistic regression model was constructed. In Model 1, only demographic characteristics and clinical baseline covariates (gender, age, Charlson comorbidity index, main diagnosis category, etc.) were included; Model 2 further incorporated the variables of hospitalization days and the proportion of various cost components to assess the incremental explanatory power of treatment process-related factors.

All statistical analyses were performed using R version 4.3.1 (R Foundation for Statistical Computing, Vienna, Austria). All statistical tests were two-tailed, and a *p* value < 0.05 was considered statistically significant.

### Descriptive statistics and stratified comparisons

Baseline characteristics of the overall analytical cohort were summarized and presented. The normality of continuous variables was assessed using the Shapiro–Wilk test. Normally distributed continuous variables are expressed as mean ± standard deviation (SD), while non-normally distributed continuous variables are presented as median with interquartile range (IQR). Categorical variables are reported as counts and corresponding percentages.

Further stratified analyses were performed by DIP classification tier and ICD-10 principal diagnosis category, respectively. The distribution of hospitalization cost components, proportion of high-magnification cases, and average LOS across each stratum were calculated. For between-group comparisons of continuous variables, one-way analysis of variance (ANOVA) was used for normally distributed data, and the Kruskal–Wallis H test was applied for non-normally distributed data. For categorical variables, between-group comparisons were conducted using the chi-square (χ^2^) test; Fisher’s exact test was used when the expected cell frequency was less than 5. Moreover, using multivariate logistic regression, the confidence intervals (CI) and odds ratios (OR) of influencing factors were calculated.

### Temporal trend analysis

Quarterly aggregated indicators, including the number of high-magnification cases and CCI per quarter, were calculated to depict temporal dynamics over the study period. Meanwhile, CCI was included as an important covariate for analysis to quantify the burden of comorbidities in patients. Subgroup analysis was conducted for the *Secondary comprehensive disease group*, with high magnification cases as the study group and non-high magnification cases as the control group. The mean differences in CCI between the two groups were compared, and the prevalence distributions of specific comorbidities such as congestive heart failure, chronic kidney disease, diabetes with target organ damage, and malignant tumors were analyzed. Continuous variables were compared between groups using the Welch two-sample t-test, and categorical variables were analyzed using the chi-square test or Fisher’s exact probability method.

Meanwhile, to explore whether there was a situation of “hospital selective admission of mild cases” among the included cases, this study analyzed the temporal trend of the case admission structure based on the burden of comorbidities during the study period.

### Bubble scatter plot analysis

Three sets of bubble scatter plots were generated to visualize the association between average hospitalization cost and the proportion of high-magnification cases across three stratification dimensions: admitting department, principal diagnosis category, and DIP classification tier. In each plot, the x-axis represents the average hospitalization cost per stratum, and the y-axis represents the proportion of high-magnification cases per stratum. Bubble size is proportional to the average CCI of each stratum, while the color gradient of bubbles corresponds to the average LOS of each stratum.

Two dashed reference lines were superimposed on each plot: a vertical line marking the overall average hospitalization cost of the entire cohort and a horizontal line marking the overall proportion of high-magnification cases. These lines divide the plot into four quadrants to facilitate stratified characterization of economic burden and high-magnification case risk across subgroups.

## Results

### Baseline characteristics of included patients under DIP payment system

Baseline characteristics of the 191,490 included patients are summarized in Table 1. Internal medicine was the dominant discharge department (43.81%), with surgery (34.87%) and gynecology (7.04%) ranking second and third. Malignant tumors, Circulatory system diseases and Urinary and reproductive system diseases were the top three principal diagnoses, accounting for 17.94, 12.72 and 8.82%, respectively. In terms of DIP grouping, *Secondary core disease category* occupied the largest share (53.46%), preceding *Primary comprehensive disease category* (27.59%) and *Tertiary core disease category* (15.37%).

**Table 1:** Basic characteristics of included inpatients under DIP payment system of an NHC-administered hospital in Guangzhou from 2024 to 2025 (*n* = 191,490).

Variable	Category/statistical indicator	Number of cases /value	Percentage (%)
Discharge departments	Surgery	66,764	34.87
	Internal medicine	83,885	43.81
	Gynecology	13,479	7.04
	Obstetrics	1,718	0.90
	Pediatrics	7,949	4.15
	Intensive care unit	3,069	1.60
	Otolaryngology	13,223	6.91
	Traditional Chinese medicine	1,403	0.73
Principal diagnosis categories	Malignant tumors	34,356	17.94
	Circulatory system diseases	24,363	12.72
	Digestive system diseases	11,948	6.24
	Respiratory system diseases	6,462	3.37
	Musculoskeletal system diseases	10,611	5.54
	Nervous system diseases	5,637	2.94
	Urinary and reproductive system diseases	16,895	8.82
	Other diseases	81,218	42.41
DIP disease categories	Tertiary core disease category	29,429	15.37
	Secondary core disease category	102,363	53.46
	Secondary comprehensive disease category	5,428	2.83
	Primary core disease category	826	0.43
	Primary comprehensive disease category	52,833	27.59
	Multi-diagnosis disease category	611	0.32
Average LOS (CNY)	Mean ± SD	6.2 ± 6.8	-
	Median [IQR‌]	4[2, 8]	-
Hospitalization costs (CNY)	Mean ± SD	27,444.7 ± 47,651	-
	Median [IQR‌]	14,323.1[7,051, 29,714]	-
Charlson Comorbidity Index	Mean ± SD	1.9 ± 2.1	-
	Median [IQR‌]	2[0, 3]	-
Medication costs (CNY)	Mean ± SD	5,036.3 ± 14,895.7	-
Consumable costs (CNY)	Mean ± SD	9,767.6 ± 26,716.2	-
High-magnification cases	Number of cases	17,929	9.36
	Average LOS (day)	9.1 ± 14.7	-
	Average hospitalization cost (CNY)	65,366 ± 131,984.7	-
	Average surplus expense (CNY)	-1,492.1 ± 7,670.7	-
	Proportion of cases with DIP deficits	-	49.42

DIP, diagnosis-intervention packet; NHC, National Health Commission; LOS, length of hospital stay; CNY, Chinese Yuan; SD, standard deviation; IQR, interquartile range.

Meanwhile, the included patients had a mean LOS of 6.2 days (median = 4.0 days) and a mean total hospitalization cost of 27,444.7 CNY (median = 14,323.1 CNY), alongside average medication, consumable and examination & laboratory costs of 5,036.3 CNY, 9,767.6 CNY and 5,493.8 CNY separately. In total, 17,929 high-magnification cases (9.36% of all cases) were screened out. The average LOS and total hospitalization cost were 1.47-fold (9.1 days) and 2.38-fold (65,366.0 CNY) higher than the overall average values, respectively. The average loss per high-magnification case was 1492.1 CNY, and 49.42% of these cases presented financial deficits.

### Hospitalization costs distribution stratified by DIP disease categories

As presented in Table 2, all indicators differed significantly among DIP subgroups (all *p* < 0.001). *Secondary core disease category* had the highest proportion of high-multiplier cases (13.11%), while *Secondary comprehensive disease category* had the largest proportion of examination and laboratory costs (41.4%). *Primary core disease category* featured the longest LOS (23.2 days), highest average standard DIP score (10,144.6), maximum hospitalization costs (141,435.6 CNY) and highest consumable cost proportion (33.2%), yet possessed the lowest share of high-magnificaiton cases (1.33%). *Multiple-diagnosis disease category* achieved the highest medication cost proportion (60.4%), while showing the shortest LOS (5.2 days), lowest average standard DIP score (1221.7) and minimal hospitalization costs (11,227.2 CNY).

**Table 2:** Cost distribution stratified by DIP disease categories in an NHC-administered hospital from 2024 to 2025.

DIP category	*n*	Average LOS (day)	Hospitalization costs (CNY)	Standard DIP scores	Medication costs (%)	Consumable costs (%)	CCI	High-magnification cases (%)
Tertiary core disease category	29,429	6.2 ± 6.7	38,237.9 ± 61,104.0	2,788.9 ± 3,497.0	11.5 ± 17.1	30.2 ± 27.5	1.6 ± 1.8	488(1.66)
Secondary core disease category	102,363	5.3 ± 5.5	23,298.2 ± 35,834.0	1,482.6 ± 1,827.5	27.1 ± 30.0	19.2 ± 21.5	2.1 ± 2.1	13,422(13.11)
Secondary comprehensive disease category	5,428	7.4 ± 7.7	22,350.0 ± 41,485.1	1,288.4 ± 1,050.8	18.3 ± 25.8	11.6 ± 16.9	1.2 ± 1.7	536(9.87)
Primary core disease category	826	23.2 ± 17.1	141,435.6 ± 135,273.8	10,144.6 ± 7,578.0	19.0 ± 18.4	33.2 ± 31.2	1.3 ± 1.4	11(1.33)
Primary comprehensive disease category	52,833	7.7 ± 8.0	28,395.4 ± 53,744.4	1,824.5 ± 1,732.2	17.3 ± 20.2	18.1 ± 48.1	1.8 ± 2.1	3,447(6.52)
Multi-diagnosis disease category	611	5.2 ± 5.9	11,227.2 ± 16,743.2	1,221.7 ± 497.8	60.4 ± 23.0	5.1 ± 7.1	3.7 ± 2.1	25(4.09)
F/χ^2^ value	-	2,039	1,489	4,014	2,437	775	610	5,127
*P* value	-	<0.001	<0.001	<0.001	<0.001	<0.001	<0.001	<0.001

DIP, diagnosis-intervention packet; NHC, national health commission; LOS, length of hospital stay; CNY, Chinese Yuan; CCI, Charlson Comorbidity Index.

### Inpatient economic burden stratified by principal diagnostic categories per ICD-10

As illustrated in Table 3, substantial inter-category heterogeneity was detected for all costs indicators across principal diagnosis categories (all *p* < 0.001). *Health status & healthcare contact factors* bore the largest fraction of high-magnification cases (36.13%), with *Blood, haematopoietic & immune disorders* (13.34%) and *Infectious & parasitic diseases* (10.75%) ranking second and third, respectively. *Infectious and parasitic diseases* were associated with the longest average LOS of 10.1 days. Conversely, *Health status & healthcare contact factors* conferred the shortest average LOS (2.6 days), alongside the dominant share of medication costs (59.6%). *Circulatory system diseases* exhibited greater values than all other diagnostic groups in two indicators including average hospitalization costs (47,090.2 CNY) and average DIP standardized score (3,085.0). By comparison, the *Pregnancy, childbirth & puerperium* group yielded the minimal average hospitalization costs (6,249.5 CNY).

**Table 3:** Cost distribution stratified by principal diagnosis categories according to ICD-10 in an NHC-administered hospital from 2024 to 2025.

Main diagnosis category	*n*	Average LOS (day)	Hospitalization costs (CNY)	Standard DIP scores	Medication costs (%)	CCI	High-magnification cases (%)
Infectious and parasitic diseases	1,954	10.1 ± 9.5	46,053.3 ± 98,158.0	2,111.6 ± 2,227.8	19.2 ± 16.4	1.8 ± 2.0	210 (10.75)
Neoplasms	50,981	7.7 ± 7.2	36,711.4 ± 40,150.6	2,608.3 ± 1,960.6	13.9 ± 15.8	2.6 ± 2.4	1,536 (3.01)
Blood, haematopoietic and immune disorders	2,332	7.4 ± 6.9	18,463.7 ± 28,412.1	1,201.5 ± 1,381.9	32.2 ± 30.6	1.7 ± 1.7	311 (13.34)
Endocrine, nutritional and metabolic diseases	7,437	5.7 ± 5.0	17,161.6 ± 25,684.7	1,063.7 ± 1,026.3	20.3 ± 29.8	2.0 ± 1.9	167 (2.25)
Nervous system diseases	5,637	7.1 ± 9.5	33,818.3 ± 73,785.3	2,487.4 ± 3,862.1	19.9 ± 30.4	0.6 ± 1.2	230 (4.08)
Circulatory system diseases	24,363	7.0 ± 7.3	47,090.2 ± 77,294.5	3,085.0 ± 3,865.1	9.4 ± 9.5	1.7 ± 1.6	852 (3.50)
Respiratory system diseases	6,462	7.4 ± 8.2	30,960.6 ± 73,707.7	1,511.8 ± 1,883.3	15.1 ± 13.6	1.3 ± 1.9	433 (6.70)
Digestive system diseases	11,948	6.2 ± 7.2	25,016.1 ± 58,435.8	1,430.6 ± 1,905.0	23.5 ± 24.4	0.9 ± 1.5	594 (4.97)
Skin and subcutaneous tissue disorders	1,988	7.9 ± 8.4	16,377.2 ± 34,685.5	887.4 ± 700.2	10.6 ± 12.2	0.7 ± 1.2	189 (9.51)
Musculoskeletal and connective tissue disorders	10,611	6.4 ± 5.4	25,630.4 ± 25,497.7	2,357.5 ± 1,831.0	12.3 ± 15.1	0.9 ± 1.3	301 (2.84)
Genitourinary system diseases	16,895	6.6 ± 5.5	17,334.7 ± 18,515.4	1,222.1 ± 961.8	13.3 ± 15.9	1.3 ± 1.6	490 (2.90)
Pregnancy, childbirth and puerperium	1,848	5.0 ± 5.7	6,249.5 ± 8,680.0	318.2 ± 213.6	7.6 ± 9.9	0.0 ± 0.3	143 (7.74)
Congenital malformations and chromosomal anomalies	3,049	7.3 ± 8.0	28,583.4 ± 40,495.6	1,870.8 ± 1,522.6	7.6 ± 7.4	0.3 ± 0.7	84 (2.76)
Symptoms, signs and abnormal findings, nec	2,518	4.4 ± 4.8	12,188.5 ± 18,096.3	769.5 ± 537.6	8.4 ± 12	0.7 ± 1.3	119 (4.73)
Injuries, poisoning and external cause sequelae	4,268	7.6 ± 8.0	31,121.1 ± 55,105.7	1,959.4 ± 1,318.7	10.9 ± 15.7	0.7 ± 1.2	308 (7.22)
Health status and healthcare contact factors	32,868	2.6 ± 2.6	10,010.0 ± 12,865.0	348.8 ± 402.7	59.6 ± 28.8	3.2 ± 2.0	11,875 (36.13)
Other diseases	6,331	5.7 ± 8.3	15,425.3 ± 28,774.0	1,283.4 ± 2,428.9	7.7 ± 10.7	0.3 ± 0.7	87 (1.37)
*F /χ^2^ value*	-	1,910	2,625	4,699	1,289	3,727	35,327
*P value*	*-*	<0.001	<0.001	<0.001	<0.001	<0.001	<0.001

DIP, diagnosis-intervention packet; NHC, national health commission; LOS, length of hospital stay; CNY, Chinese Yuan; ICD-10, international classification of diseases-10th revision; CCI, Charlson Comorbidity Index.

### Trends in hospitalization costs and the number of high-magnification cases

**Figure 1:**
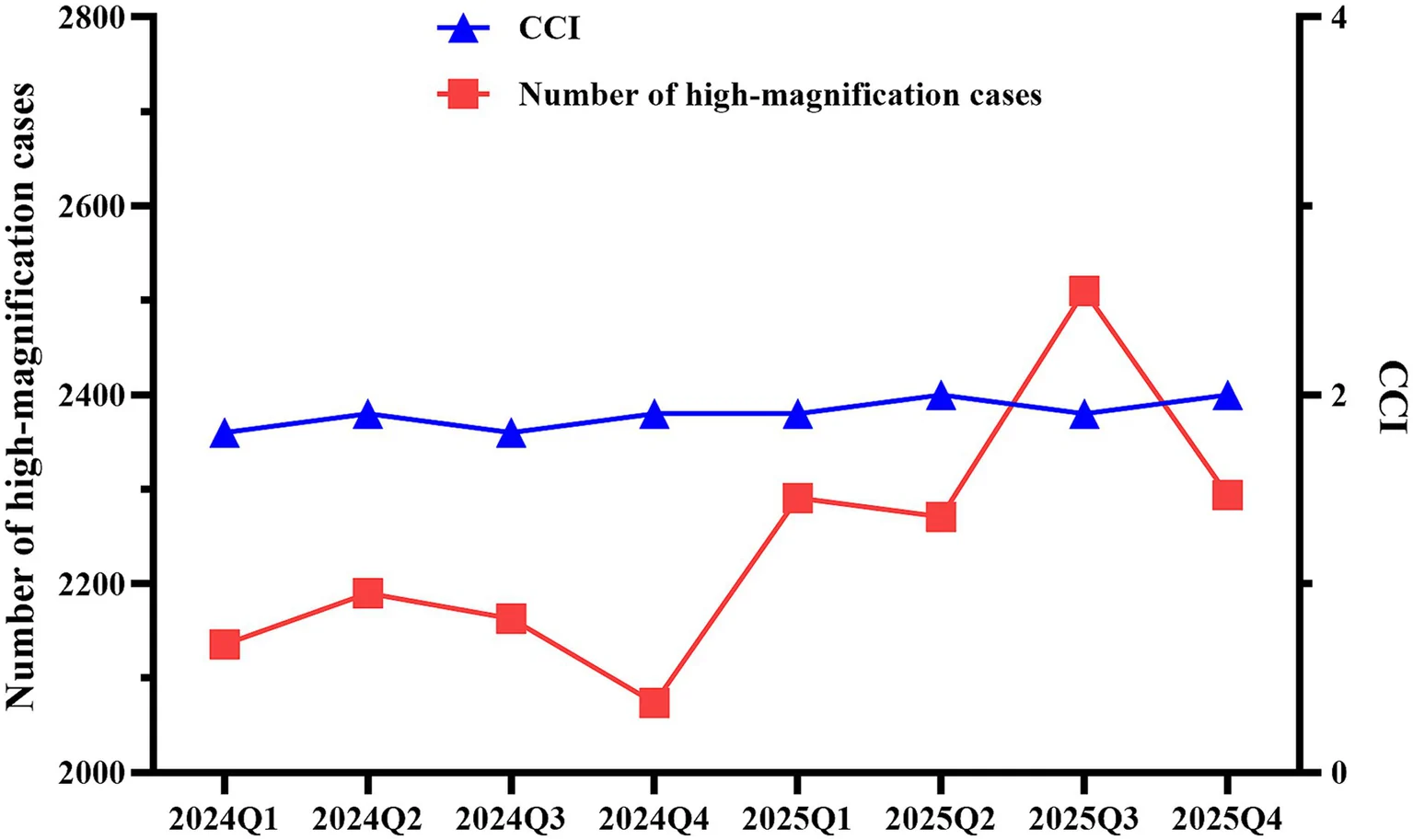
Quarterly trends of average Charlson Comorbidity Index and the number of high-magnification cases from 2024 to 2025. The blue solid line represents the average Charlson Comorbidity Index (right Y-axis), and the red solid line represents the number of high-magnification cases (left Y-axis). CCI: Charlson Comorbidity Index.

Figure 1 is a dual-axis line chart depicting the trend of CCI and the number of high- magnification cases over a period of 8 quarters from Q1 2024 to Q4 2025. The CCI indicator has remained stable overall, fluctuating slightly within the range of 1.8 to 2.0 and showing a slow upward trend, indicating that the burden of comorbidities in the sample patients is basically stable, while the number of high-magnification cases fluctuates significantly. In 2024, it hovered between 2,100 and 2,200 cases and reached its lowest point in Q4. Starting from 2025, it significantly increased, reaching a peak of approximately 2,510 cases in Q3 and then falling back to 2,294 cases in Q4. The two trends are not completely synchronized, suggesting that the increase in high-magnification cases may not be solely driven by the severity of patients’ conditions, but may be related to other factors.

### Stratified multi-dimensional distributions of high-magnification cases

**Figure 2:**
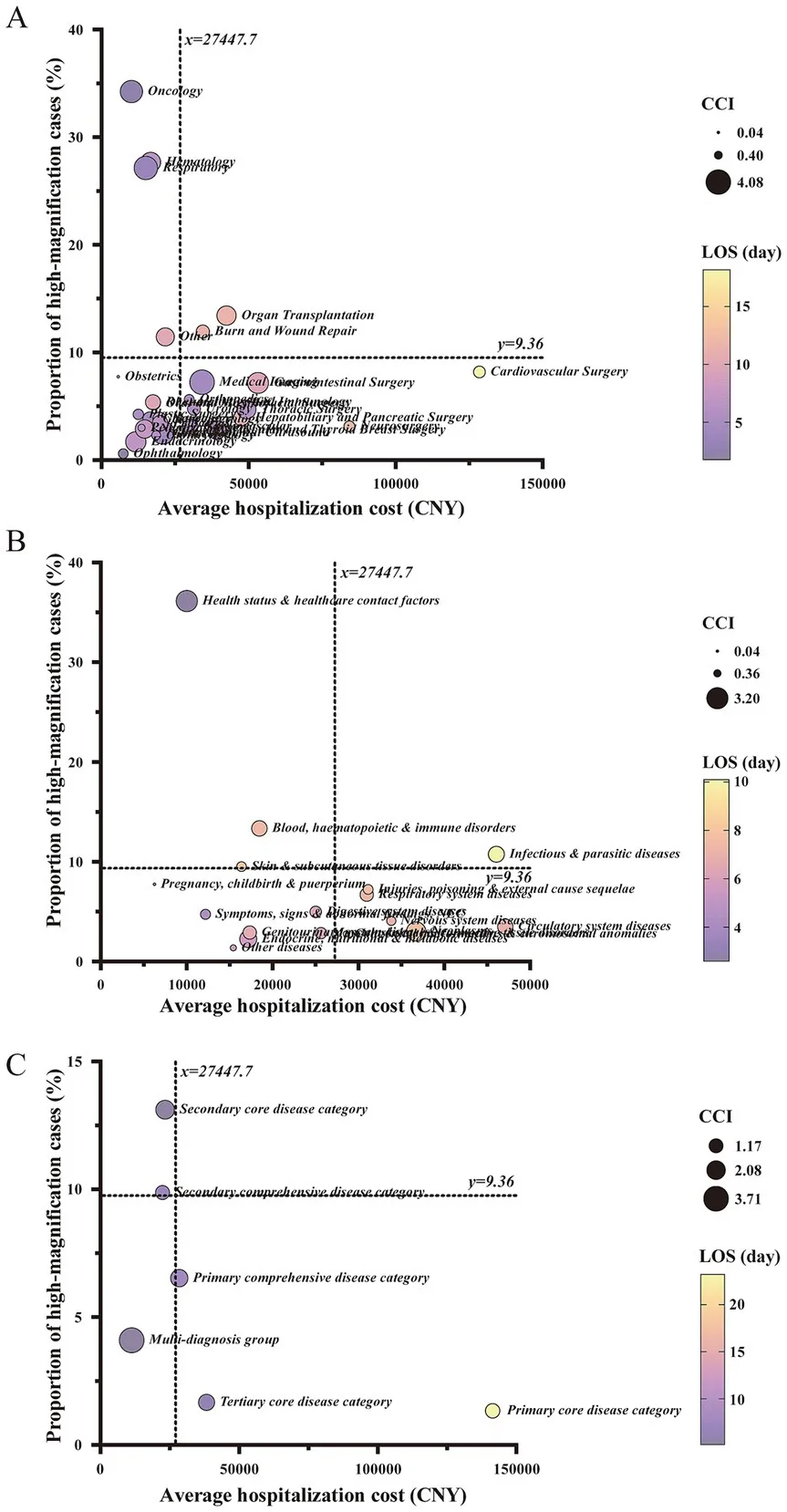
Bubble charts illustrating the proportion of high-magnification cases across three classification dimensions. **(A)** By clinical department/surgical specialty. **(B)** By ICD-10 disease system category. **(C)** By DIP disease categories. The *x*-axis represents average hospitalization cost (CNY); the *y*-axis represents the proportion of high-magnification cases (%). Bubble size denotes the Charlson Comorbidity Index (CCI), with larger bubbles indicating greater comorbidity burden; bubble color represents length of stay (LOS), with warmer colors indicating longer hospital stays. The vertical dashed line (*x* = 27,447.7 CNY) and horizontal dashed line (*y* = 9.36%) indicate the respective mean values, dividing each panel into four quadrants.

Figure 2 shows the distribution of high-magnification cases across three classification dimensions, presented in the form of bubble charts, and compares the proportion of high-magnification cases with the average hospitalization cost. In all panels, the size of the bubbles represents the CCI, and the color of the bubbles indicates the length of hospital stay (LOS), with warmer colors representing longer hospital stays. The figure includes two reference lines: a vertical dashed line at x = 27,447.7 yuan (the average hospitalization cost threshold), and a horizontal dashed line at y = 9.36% (the average proportion of high-magnification cases).

Figure 2A stratifies the cases by clinical departments. The proportion of high-magnification cases is the highest in the department of *Oncology* (34.24%), followed by the *Hematology* (27.67%), with both having relatively lower average costs (both below 20,000 CNY). The proportions of high-magnification cases are also high in *Burn and Wound Repair* (11.93%) and *Organ Transplantation* (11.66%), but the average costs are at a medium level. The average cost of *Cardiovascular Surgery* is the highest (128,483.5 CNY), but the proportion of high-magnification cases is moderate (8.18%). Most departments are concentrated in the lower left quadrant, with their average costs below 50,000 CNY, and the proportion of high-magnification cases less than 10%.

Figure 2B classifies the cases by ICD-10. *Health status & healthcare contact factors* show that the proportion of high-magnification cases is the highest (36.13%), with an average cost of 10,010.04 CNY. The proportions of high-magnification cases for *Blood, haematopoietic & immune disorders* and *Skin & subcutaneous tissue disorders* are 13.34 and 9.51%, respectively. Among all disease categories, the average cost of *Circulatory system diseases* is the highest (47,090.19 CNY), with a high-magnification case proportion of 3.5%. Most disease systems are below the average threshold.

Figure 2C classifies the cases by DIP disease categories. The proportion of high-magnification cases in the *Secondary core disease category* is the highest (13.11%), followed by the *Secondary comprehensive disease category* (9.87%), both below 24,000 CNY. The average cost of the *Primary core disease category* is the highest (141,435.57 CNY), but the proportion of high-magnification cases is the lowest (1.33%). Notably, the *Multi-diagnosis disease category* has a relatively lower average cost (11,227.22 CNY), but its proportion is relatively low (4.09%); while the *Tertiary core disease category* has a medium-level cost (38,237.86 CNY), but a relatively low proportion (1.66%).

Consequently, Table 4 interprets the representative department/disease categories in each quadrant of Figure 2 and their corresponding management suggestions. Overall, high-magnification cases are more concentrated in specific specialties and disease categories than in high-cost categories, suggesting that in DIP payment, the drivers of cost increase and abnormal conditions differ.

**Table 4:** Management quadrant matrix of DIP case types by high-magnification cases proportion and hospitalization cost level.

High-magnification cases proportion/cost	Low cost	High cost
High-magnification proportion	Q1 (High proportion · Low cost) Representative categories: A: Oncology, Hematology B: Health status factors, Blood/immune C: Secondary core disease category Management focus: Coding accuracy, Comorbidity adjustment	Q2 (High proportion · High cost) Representative categories: A: Organ transplantation, Burn repair B: Infectious and parasitic diseases Management focus: Cost control, Pathway optimization
Low-magnification proportion	Q3 (Low proportion · Low cost) Representative categories: A: Most medical/surgical departments B: Pregnancy, Digestive, Endocrine C: Multi-diagnosis group Management focus: Routine monitoring	Q4 (Low proportion · High cost) Representative categories: A: Cardiovascular surgery B: Circulatory, Cerebrovascular C: Primary core disease category Management focus: Case-level review, Justification

DIP, Diagnosis-Intervention Packet. Cases are cross-classified by their share of total case volume (case proportion: high vs. low) and by average resource consumption (cost: low vs. high). Quadrants are defined accordingly: Q1 = high proportion and low cost; Q2 = high proportion & high cost; Q3 = low proportion and low cost; Q4 = low proportion and high cost. Representative categories (A/B/C) list the leading clinical departments, disease-factor groups, and core disease categories falling within each quadrant, based on hospital DIP data. “Management focus” indicates the recommended priority strategy for cases in that quadrant. Color shading stratifies management priority: cool tones (green, blue) denote quadrants with contained cost burden requiring routine or quality-focused management; warm tones (orange) denote quadrants requiring active cost control or case-level justification. Saturation within warm-toned quadrants distinguishes aggregate-cost-driven (Q2) from per-case-cost-driven (Q4) priorities.

### Multivariate logistic regression analysis of influencing factors for high-magnification cases

Multivariate logistic regression analyses are summarized in Table 5. Following adjustment for fixed departmental confounders, LOS (OR = 1.139, 95% CI 1.136–1.143, *p* < 0.001), consumable cost proportion (OR = 1.029, 95% CI 1.026–1.031, p < 0.001), medication cost proportion (OR = 1.053, 95% CI 1.051–1.055, p < 0.001), examination & laboratory cost proportion (OR = 1.009, 95% CI 1.007–1.011, *p* < 0.001), and age (OR = 1.006, 95% CI 1.004–1.007, p < 0.001) emerged as independent positive predictors of high-magnification cases. Each 1% increment in the proportion of consumable costs, medication costs, and examination & laboratory costs was associated with a 2.9, 5.3, and 0.9% elevation in the odds of high-magnification cases, respectively. Meanwhile, taking *Tertiary core disease category* as the reference group, *Secondary comprehensive disease category* exhibited significantly higher odds of high- magnification cases (OR = 9.474, 95% CI 8.028–11.180, *p* < 0.001), whereas *Primary core disease category* presented markedly lower odds (OR = 0.011, 95% CI 0.005–0.027, *p* < 0.001). Furthermore, taking *Other disease*s as the reference, *Pregnancy, childbirth & puerperium*, *Health status & healthcare contact factors* and *Skin & subcutaneous tissue disorders* subgroups demonstrated substantially elevated risks of high-magnification cases (*p* < 0.001), with corresponding OR values of 8.162, 7.292 and 3.175, respectively. In addition, quarterly odds declined consecutively from Q1 2024 to Q4 2025. Gender exerted no statistically significant effect (*p* > 0.05).

**Table 5:** Multivariate logistic regression analysis of the influencing factors for high-magnification risk under DIP payment system.

Independent variable	OR	95% CI	*t*	*P* value
Constant term	0.001	0–0.001	−22.330	<0.001
Medication cost (%)	1.053	1.051–1.055	54.472	<0.001
Consumable cost (%)	1.029	1.026–1.031	22.815	<0.001
Examination and laboratory cost (%)	1.009	1.007–1.011	7.696	<0.001
LOS (days)	1.139	1.136–1.143	84.055	<0.001
Age (years)	1.006	1.004–1.007	8.696	<0.001
Gender (male = 1, female = 0)	0.977	0.937–1.019	−1.099	0.272
CCI	1.068	1.057–1.08	12.273	<0.001
DIP category (Taking the three-level core disease category as the control group)				
Secondary core disease category	3.625	3.183–4.129	19.405	<0.001
Secondary comprehensive disease category	9.474	8.028–11.180	26.617	<0.001
Primary core disease category	0.011	0.005–0.027	−9.963	<0.001
Primary comprehensive disease category	5.421	4.79–6.136	26.761	<0.001
Multi-diagnosis disease category	0.133	0.086–0.206	−9.033	<0.001
Main diagnosis category (Taking other diseases as the control group)				
Infectious and parasitic diseases	0.761	0.550–1.052	−1.651	0.099
Neoplasms	0.481	0.368–0.629	−5.360	<0.001
Blood, haematopoietic and immune disorders	1.241	0.920–1.674	1.412	0.158
Endocrine, nutritional and metabolic diseases	0.445	0.325–0.608	−5.068	<0.001
Nervous system diseases	0.467	0.343–0.635	−4.862	<0.001
Circulatory system diseases	0.682	0.516–0.901	−2.696	0.007
Respiratory system diseases	1.092	0.815–1.463	0.589	0.556
Digestive system diseases	0.902	0.683–1.191	−0.728	0.467
Skin and subcutaneous tissue disorders	3.175	2.322–4.341	7.239	<0.001
Musculoskeletal and connective tissue disorders	0.837	0.626–1.120	−1.197	0.231
Genitourinary system diseases	1.071	0.810–1.415	0.480	0.631
Pregnancy, childbirth and puerperium	8.162	4.503–14.793	6.919	<0.001
Congenital malformations and chromosomal anomalies	0.687	0.476–0.991	−2.007	0.045
Symptoms, signs and abnormal findings, nec	2.124	1.528–2.951	4.488	<0.001
Injuries, poisoning and external cause sequelae	1.437	1.068–1.931	2.399	0.016
Health status and healthcare contact factors	7.292	5.575–9.537	14.508	<0.001
Quarter (Taking the 2024Q1 as the control group)				
2024Q2	1.219	1.126–1.32	4.892	<0.001
2024Q3	1.160	1.071–1.256	3.663	<0.001
2024Q4	1.189	1.097–1.289	4.200	<0.001
2025Q1	1.173	1.084–1.269	3.945	<0.001
2025Q2	1.053	0.972–1.139	1.269	0.204
2025Q3	1.095	1.013–1.183	2.281	0.023
2025Q4	1.070	0.988–1.159	1.671	0.095
Fixed effect of the department category	Controlled	-	-	-

DIP, Diagnosis-Intervention Packet; LOS, length of hospital stay; OR, odds ratios; CI, confidence interval; CCI, Charlson Comorbidity Index.

In addition, as show in Supplementary Table S1, the sensitivity analysis results showed that under the 5 critical value conditions (2.0–4.8 times), the effect direction and statistical significance of the core independent variables remained stable. The increase in the proportion of drug expenses (OR: 1.046–1.059, all *p* < 0.05), the extension of hospital stay (OR: 1.116–1.139, all *p* < 0.001), the increase in the Charlson comorbidity index (OR: 1.068–1.097, all p < 0.001), and the classification as the *Secondary comprehensive disease group* (OR: 9.47–15.25, all p < 0.001) were all significantly positively associated with the high-magnification case determination. It can be seen that the risk factors identified in this study for high-magnification cases have good robustness, and the conclusion is not dependent on the selection of specific thresholds.

Furthermore, as shown in Supplementary Table S2, the corrected VIF values for all independent variables ranged from 1.00 to 1.61, all below the critical value of 2.0, indicating that there is no significant multicollinearity problem in the regression model. In addition, as shown in Supplementary Table S3, Model 1, which only included the baseline characteristics of patients, showed that demographic indicators and clinical comorbidity indicators were significantly related to the determination of high-risk cases: for every 1-point increase in the CCI, the risk of high-magnification cases increased by 16.8% (OR = 1.168); the high-magnification risk in the ICD group of *Health status & healthcare contact factors* was the highest (OR = 24.42). After adding the variables of cost composition and hospital stay days to Model 2, the model fitting effect significantly improved (McFadden pseudo R^2^ increased from 0.244 to 0.430; Nagelkerke R^2^ increased from 0.307 to 0.509), suggesting that factors related to the medical treatment process can further explain some of the variation sources of high-risk cases beyond the baseline disease characteristics of patients.

As shown in Supplementary Table S4, within the *Secondary comprehensive disease* group, the comorbidity burden of high-ratio cases was significantly higher than that of non-high-ratio cases: the mean CCI value of the high-ratio cases group was 1.77 ± 1.45, while that of the non-high-ratio cases group was 1.13 ± 1.28. The difference between the two groups was statistically significant (Welch two-sample t-test: t = −5.35, df = 379.8, *p* < 0.001; 95% confidence interval of mean difference: 0.41–0.88). Multivariate regression analysis showed that the *Secondary comprehensive disease* group was the strongest risk factor for high-ratio cases (OR = 9.47, 95% CI: 8.03–11.18); sensitivity analysis further confirmed that within the entire threshold range of 2.0–4.8 times, this group consistently maintained the highest high-ratio risk (OR range: 9.47–15.25), and its high-risk characteristics remained stable.

As shown in Supplementary Table S5, the proportion of hospitalized cases with a high burden of comorbidities (CCI ≥ 3) increased from 25.7% in the first quarter of 2024 to 30.9% in the fourth quarter of 2025, showing a significant upward trend (Cochran-Armitage trend test: Z = −12.78, *p* < 0.001), which does not support the inference that the hospital deliberately avoided admitting complex critically ill patients.

## Discussion

As a locally developed case-mix payment model underpinned by big data, DIP represents a core component of China’s medical insurance payment reform. Operating under a regional global budget framework with standardized disease-specific payment groups, the DIP system is designed to incentivize healthcare institutions to proactively optimize resource allocation and curb the irrational growth of medical expenditures (1). Nevertheless, amid the nationwide rollout of the reform, high-magnification cases, defined as inpatient episodes whose actual medical costs substantially exceed the corresponding standard DIP payment threshold, have emerged as a pressing operational challenge for tertiary hospitals. Despite accounting for a relatively small proportion of total admissions, these cases consume a disproportionate share of medical resources, and have exposed fundamental limitations in current DIP grouping algorithms, most notably inadequate payment coverage for patients with complex and severe clinical conditions (7, 11).

This retrospective observational study was conducted based on inpatient data from a tertiary referral hospital directly affiliated with the National Health Commission in southern China, to comprehensively analyze the epidemiological characteristics, economic burden, and independent influencing factors of high-magnification cases under the DIP payment system. A total of 191,490 inpatients admitted between 2024 and 2025 were included. The results showed that high-magnification cases accounted for only 9.36% of total admissions, but their average hospitalization cost was 2.38 times the cohort average. Furthermore, 49.42% of high-magnification cases were associated with varying degrees of payment deficit, a pattern highly consistent with the operational impact of extreme-cost cases under the international DRG payment system (2, 3). Multivariate logistic regression analysis showed that prolonged LOS, increased proportion of medical consumable costs, increased proportion of medication costs, comorbidities, diagnosis with pregnancy-related diseases, and classification into *Secondary comprehensive disease category* were independent risk factors for high-magnification cases.

As a large-sample real-world study conducted in a top-tier tertiary hospital in southern China, these findings complement empirical evidence on the operational outcomes of DIP reform in tertiary hospitals in developed regions of China, and provide data support for the refined management of high-magnification cases and optimization of medical insurance policies in similar institutions.

## Characteristics, influencing factors and current status of high-magnification cases

### Incidence and burden characteristics of high-magnification cases

The overall incidence of high-magnification cases in this study was 9.36%, which falls within the range reported in similar domestic and international studies. Previous research on DRG payment systems has shown that extreme-cost cases typically account for 2 to 5% of total admissions, with the exact proportion closely related to the complexity of cases treated by the hospital and the threshold set for payment standards (12). Empirical studies on DIP pilot sites in China have also found that tertiary hospitals, which treat a higher proportion of complex and severe cases, generally have a higher incidence of high-magnification cases and greater cost overshoot compared with secondary healthcare institutions (13).

As a tertiary care center for complex and severe diseases in South China, the study hospital admits a high proportion of transferred patients with complex conditions. Accordingly, the average cost of the high-magnification cases was 2.38 times that of the overall average cost in this study. This situation is consistent with the results of specialized studies: complex procedures such as major pancreatic surgery and surgery for male reproductive system malignancies often incur actual resource consumption that significantly exceeds standardized payment amounts, and represent high-incidence areas for high-magnification cases (7, 11). Furthermore, the finding that 49.42% of high-magnification cases are associated with payment deficits aligns with international research conclusions: under prospective payment systems, a small number of extreme-cost cases contribute disproportionately to hospitals’ financial risk and represent one of the main sources of operational losses (2).

### Significant impact of length of stay on high-magnification risk

Consistent with the conclusions of most DIP and DRG-related studies worldwide, this study confirms that prolonged length of stay is the strongest modifiable predictor of high-magnification events, with each additional day of hospitalization associated with a 13.9% increase in the risk of cost overrun. The underlying mechanism for this association operates on two levels. On one hand, extended length of stay directly accumulates direct medical costs such as ward nursing, routine examinations, and basic medications. On the other hand, prolonged hospitalization increases the risk of adverse events such as nosocomial infections and complications, which further drive additional medical resource consumption (14). A DRG-based study on ischemic stroke also confirmed that length of stay is the core driver of fluctuations in hospitalization costs, and reducing average length of stay is a key target for controlling case cost overruns (15).

This finding also validates the cost-control value of clinical pathway optimization. Previous studies have successfully reduced average length of stay and lowered per-case costs without compromising medical quality by constructing a “double helix model” of cost accounting and pathway optimization (6). A study on elective benign gynecological surgery also showed that clinical pathway optimization under the Enhanced Recovery After Surgery (ERAS) concept can simultaneously achieve shortened hospital stays and cost control without impairing patient prognosis (16). Similarly, a study on uvulopalatopharyngoplasty in patients with obstructive sleep apnea confirmed that proactive clinical pathway optimization can significantly reduce pharmaceutical and consumable costs and shorten hospitalization duration without compromising patient safety or long-term surgical outcomes (17).

### Driving role of medical consumables in cost overruns

An increased proportion of medical consumable costs represents another key risk factor for high-magnification cases, and this characteristic is particularly prominent in tertiary care centers for complex and severe diseases with a high volume of surgical procedures. With the advancement of the national centralized drug procurement policy, the growth of pharmaceutical costs has been effectively curbed, whereas high-value medical consumables have gradually become the main driver of growth in hospitalization costs. An interrupted time series study by Zhu et al. (5) conducted in a tertiary hospital in China showed that after the implementation of DIP payment reform, the proportion of consumable costs in surgical patients increased significantly, contributing 55.83% to changes in cost structure and representing the primary cause of cost overruns in surgical cases.

Subgroup analysis in this study further supports this conclusion: *Pregnancy, childbirth & puerperium* group was associated with the highest risk of high-magnification cases (OR = 8.162, *p* < 0.001). The core reason for this is that the rescue of obstetric emergencies such as postpartum hemorrhage and amniotic fluid embolism requires extensive use of interventional consumables and high-value hemostatic materials, resulting in actual costs far exceeding the standard DIP payment benchmark. A study on the implementation effect of DRG in obstetrics departments of tertiary hospitals in South Korea also found that emergency obstetric surgeries have a high proportion of consumable costs and represent a concentrated area of high-cost cases; furthermore, rigid cost control may lead hospitals to selectively reduce high-risk emergency surgeries, indirectly compromising medical quality (18). In addition, studies on pancreatic surgery and urologic oncology surgery have similarly confirmed that the use of consumables such as robotic-assisted systems and high-value implants in complex surgeries is a core driver of case cost overruns (7, 11).

### Structural shortcomings of comprehensive DIP groups

Notably, this study found that classification into *Secondary comprehensive disease category* was associated with the highest risk of high-magnification cases (OR = 9.474, *p* < 0.001). This finding reveals structural shortcomings in current DIP grouping rules that have rarely been highlighted in previous research. Comprehensive DIP groups were originally designed to accommodate complex cases with multiple comorbidities that cannot be classified into core disease groups. However, current grouping rules have relatively coarse granularity and fail to fully incorporate weight adjustments for comorbidities and complications. As a result, grouping outcomes cannot accurately reflect the actual resource consumption of complex cases, creating a structural paradox in which “the more complex the condition, the more severe the deficit.”

This issue was also prevalent during the development of international DRG systems: early DRG groups in Germany had insufficient homogeneity, leading to inadequate payment compensation for complex patients with multiple underlying diseases, which to some extent induced hospitals to avoid treating severely ill patients (2). Studies on conditions such as fractures and chronic kidney disease have also confirmed that comorbidities such as hypertension and diabetes significantly increase hospitalization costs, and the failure of current grouping rules to account for such individual differences is an important cause of cost overruns in comprehensive groups (19, 20). To address this issue, scholars have proposed an adaptive and interpretable case grouping framework, which refines comprehensive disease groups by introducing comorbidity adjustment factors to improve the match between payment standards and actual costs, providing a technical path for the optimization of grouping rules (21).

### Trend of the number of high-magnification cases and the management bottlenecks

Theoretically, the quarterly decline in high-risk cases might be caused by hospitals selectively admitting patients with mild symptoms. However, the proportion of severe cases with complications continued to increase during the same period, which does not conform to the expected outcome of the skimming admission hypothesis. Therefore, while maintaining a cautious attitude, we believe that this downward trend can be partly attributed to the refined operational management and control of hospitals. Meanwhile, we acknowledge that there may still be unaccounted confounding factors that could affect the trend change. International studies have also confirmed that big data-based proactive cost warning systems can effectively identify high-cost risk cases, reduce the incidence of cost overruns through early intervention, and avoid negative impacts on medical quality. For example, a study on intensive care unit (ICU) patients constructed a risk stratification model using machine learning algorithms, which can accurately identify inpatients with high needs and high costs at the early stage of admission, providing tool support for proactive cost management (22). A study on severe COVID-19 patients also verified the value of logistic regression models in predicting high-cost cases, which can provide a basis for refined cost management (23).

However, it should be noted that there are still significant gaps in handling individual complex and severe cases, indicating that relying solely on administrative control measures has bottlenecks when dealing with extremely complex cases. This phenomenon is consistent with warnings from previous research: a one-size-fits-all restrictive approach to high-magnification cases will dampen hospitals’ enthusiasm for treating patients with complex and severe conditions, which is not conducive to the improvement of disciplinary capabilities and runs counter to the functional positioning of tertiary hospitals as regional centers for severe disease care (10). A study in the field of pediatrics showed that after DIP reform, both the proportion and absolute number of complex/critical cases showed a downward trend, which to some extent reflects hospitals’ adjustment of admission structure to avoid high-magnification risks (9).

## Practical implications for clinical management and policy

### Implications for hospital operational management

For hospital operational managers, firstly, targeted and precise cost control measures should be implemented for high-risk departments and disease types, with a focus on standardizing the rational use of high-value medical consumables, while improving the efficiency of length-of-stay management through clinical pathway optimization. Existing research evidence shows that developing differentiated clinical pathways for diseases with high incidence of high-magnification cases, eliminating unnecessary examination and medication links while retaining essential advanced diagnostic and therapeutic technologies, can effectively reduce the risk of cost overruns without compromising medical quality (17). Studies on different types of surgery have also confirmed that optimizing clinical pathways and consumable management according to disease characteristics can significantly reduce the proportion of case cost overruns (24). Meanwhile, the role of clinical pharmacists in medication therapy management should be fully utilized to standardize drug use through measures such as optimizing anti-infective regimens, further reducing the proportion of drug costs and irrational expenditures (25).

On this basis, a hierarchical classification management mechanism for high-magnification cases should be established, with differentiated management strategies adopted according to the cause and nature of cost overruns: avoidable cost overruns caused by non-standard diagnosis and treatment should be subject to strict management and performance constraints; systemic cost overruns caused by unreasonable grouping rules should be submitted by the medical insurance management department to the medical insurance agency for unified adjustment; necessary high-value cost overruns generated by the application of new technologies and the treatment of acute and critical illnesses should be included in the category of disciplinary development support and excluded from routine performance assessment, so as to guarantee the capacity for severe disease diagnosis and treatment and the momentum of disciplinary innovation. International studies have also pointed out that classified management of high-magnification cases, distinguishing between controllable and uncontrollable factors, can maintain hospitals’ capacity for severe disease diagnosis and treatment and technological innovation vitality while controlling costs, which is in line with the development direction of value-based healthcare (26, 27).

Secondly, an intelligent early warning system for high-magnification cases based on multi-source data should be constructed, integrating patients’ demographic characteristics, diagnostic information, treatment plans, and real-time cost data, to identify high-magnification cases at the early stage of admission and initiate proactive intervention. At present, multiple studies have verified the value of prediction models in identifying high-cost cases: studies on ICU patients have constructed risk stratification models through machine learning algorithms, which can accurately identify inpatients with high demand and high cost (22, 23); studies on diseases such as unstable angina pectoris and chronic kidney disease have also confirmed that individual characteristics such as age, number of comorbidities, and admission status are important predictors of hospitalization costs, which can be incorporated into early warning models to improve identification accuracy (20, 28). Through pre-warning and multi-disciplinary intervention, traditional post-event cost control can be transformed into pre-event risk prediction and in-event process intervention, improving the initiative and precision of management.

Thirdly, the quality of medical record coding should be continuously improved to ensure the accuracy of DIP grouping. Accurate diagnosis and procedure coding are prerequisites for ensuring correct case grouping and reasonable reimbursement. Coding deviations may lead to cases being classified into groups with lower payment standards, artificially increasing the incidence of high-magnification cases. International experience shows that the quality of medical record coding directly affects the accuracy of DRG/DIP grouping and payment outcomes, and coding errors are an important avoidable factor leading to unreasonable hospital losses (2, 21). Therefore, hospitals should strengthen the training of coding staff on clinical knowledge and rule updates, establish a two-way communication mechanism between clinicians and coders, and improve whole-process coding quality control, so as to reduce operational losses caused by coding errors.

### Recommendations for medical insurance policy optimization

For medical insurance administrative departments, firstly, a formal payment adjustment mechanism for abnormal high-magnification cases should be established to provide additional compensation for reasonable cases with extremely high costs, so as to reasonably share the risk of cost overruns between the medical insurance fund and hospitals, and avoid excessive rigid cost control forcing down medical quality. International experience in payment reform shows that prospective payment without risk sharing will induce a series of ethical and quality problems, including hospitals shunning patients with complex clinical conditions and socially vulnerable groups, compressing necessary diagnosis and treatment links to affect patient prognosis, and shifting costs to patients’ out-of-pocket payments (29, 30). Empirical studies on the Chinese population also show that the 30-day readmission rate of low-income groups has increased after DIP reform, suggesting that rigid cost control may have a negative impact on the medical quality of vulnerable group (31). Studies on patients with coronary heart disease and lung cancer also found that there is a certain degree of cost-shifting behavior after DIP reform, with increased out-of-pocket expenses and the proportion of out-of-pocket payments for Class B drugs (32, 33). Therefore, establishing a scientific exemption and supplementary payment mechanism for abnormal cases is a necessary supporting measure to ensure medical quality and equity (34).

Secondly, the DIP grouping algorithm should be optimized, and the granularity of comprehensive disease groups should be refined by introducing adjustment factors for comorbidities and complications, so as to make payment standards closer to actual clinical resource consumption. As mentioned above, the insufficient homogeneity of current comprehensive DIP groups is the core institutional cause of structural deficits (2). Future policy optimization can draw on the evolution experience of the international DRG system, integrate clinical expert demonstration on the basis of big data grouping, increase the weight assignment for cases with complex comorbidities, and narrow the gap between payment standards and actual costs. The adaptive and interpretable case grouping framework also provides a technical reference for grouping optimization, which can improve the rationality of complex case grouping while retaining the advantages of DIP big data (21).

Thirdly, dynamic payment adjustments should be implemented for special disease types with high diagnosis and treatment difficulty and high proportion of consumable costs, such as acute and critical obstetric diseases, hematological diseases, and malignant tumors. A fast-track access and score adjustment mechanism for new technologies and projects should be established to provide reasonable compensation for tertiary hospitals’ function of diagnosing and treating difficult and severe diseases. As regional medical highlands, tertiary hospitals undertake the core functions of emergency and critical care treatment and medical technology innovation, and the complex cases they treat naturally have higher resource consumption attributes (4, 13). If payment standards cannot dynamically match the development of clinical technology and changes in disease spectrum, it will force hospitals to reduce the development of high-difficulty and high-risk diagnosis and treatment projects, ultimately damaging patients’ access to diagnosis and treatment and the overall regional medical level (9, 24).

In addition, attention should be paid to the issue of cost equity among patients with different types of medical insurance. Existing studies show that there are differences in the concentration of hospitalization costs among patients with different types of medical insurance, and this difference is particularly prominent in tertiary hospitals (35). Studies on patients with type 2 diabetes and chronic obstructive pulmonary disease have also confirmed that DIP reform has heterogeneous impacts on the cost burden of different medical insurance groups (36, 37). Therefore, population differences should be fully considered in the policy-making process to avoid payment reform exacerbating medical inequity.

There were several potential limitations in this study. Firstly, this is a single-center study with data derived from a top national tertiary referral hospital that admits a high proportion of transferred patients with complex and difficult conditions. The case structure differs significantly from that of ordinary tertiary hospitals and secondary hospitals, which limits the generalizability of the study findings. Secondly, as a retrospective study based on routinely collected administrative and medical record data, coding bias cannot be completely eliminated, and the accuracy of medical record coding directly affects DIP grouping results and the identification of high-magnification cases. Thirdly, this study did not include clinical outcome indicators such as in-hospital mortality rate, 30-day readmission rate, and incidence of medical complications. As a result, it was unable to comprehensively evaluate the impact of current cost control measures on medical quality, nor could it deeply analyze the balance relationship between cost control and quality assurance. Future studies should expand the sample size to cover medical institutions of different levels and regions, and integrate clinical outcome indicators to construct a three-in-one comprehensive evaluation system of “cost-efficiency-quality,” so as to more comprehensively evaluate the comprehensive effects of DIP reform.

## Conclusion

This study indicates that under the DIP payment system, prolonged hospitalization time, increased proportion of high-value medical consumables and pharmaceutical costs, insufficient granularity of some DIP groups, and specific types of severe cases are the core reasons for the cost overrun of cases. By improving the payment mechanism for abnormal cases, optimizing disease grouping rules, and dynamically adjusting the payment standards for specific types of severe cases, forming an institutional synergy, it is conducive to achieving a win-win situation where multiple parties can ensure the quality of nursing while improving the efficiency of medical security fund utilization and maintaining the stable operation of hospitals.

## Data Availability

The raw data supporting the conclusions of this article will be made available by the authors, without undue reservation.
