# Supplementary material for: High-magnification inpatient cases under DIP payment reform: prevalence, cost burden, and risk factors in an NHC-administered hospital in China
Source: Front Public Health. 2026 Sep 3;14:1915516. doi: 10.3389/fpubh.2026.1915516 (PMC13581624; doi:10.3389/fpubh.2026.1915516)
Supplement: Supplementary file 1 [file Table_1.docx]

**Table S1. Sensitivity analysis of multivariable logistic regression for DIP high-magnification cases under alternative cost-ratio thresholds** (2.0, 2.5, 3.0, 3.1, and 4.8).

| **Risk factor** | **OR^(threshold = 2.0)^** | **OR^(threshold = 2.5)^** | **OR^(threshold = 3.0)^** | **OR^(threshold = 3.1)^** | **OR^(threshold = 4.8)^** | **Direction consistency** |
| --- | --- | --- | --- | --- | --- | --- |
| Drug-cost ratio | 1.053 | 1.058 | 1.059 | 1.057 | 1.046 | Consistent (all >1, all significant) |
| Length of stay | 1.139 | 1.138 | 1.132 | 1.131 | 1.116 | Consistent (all >1, all significant) |
| Age | 1.006 | 1.007 | 1.008 | 1.008 | 1.011 | Consistent (all >1, all significant) |
| CCI | 1.068 | 1.080 | 1.094 | 1.096 | 1.097 | Consistent (all >1, all significant) |
| Secondary comprehensive disease group | 9.474 | 13.004 | 14.779 | 15.251 | 14.300 | Consistent (all >1, all significant) |
| Secondary core disease group | 3.625 | 3.842 | 3.689 | 3.661 | 3.684 | Consistent (all >1, all significant) |
| Primary comprehensive disease group | 5.421 | 6.281 | 6.792 | 6.943 | 8.583 | Consistent (all >1, all significant) |

*A DIP high-rate case was defined as an inpatient encounter whose actual DIP score exceeded the standard score by the indicated multiple. All odds ratios were statistically significant (P < 0.05) under every threshold tested, confirming the stability of the identified risk factors. OR, odds ratio; DIP, Diagnosis-Intervention Packet.*

**Table S2. Variance inflation factor diagnostics for multicollinearity among predictors in the multivariable logistic regression model.**

| **Predictor** | **GVIF** | **df** | **GVIF^1/(2×df)^** |
| --- | --- | --- | --- |
| Drug-cost ratio | 2.59 | 1 | 1.61 |
| Material-cost ratio | 1.40 | 1 | 1.18 |
| Laboratory/examination-cost ratio | 1.98 | 1 | 1.41 |
| Length of stay | 1.15 | 1 | 1.07 |
| Age | 1.45 | 1 | 1.20 |
| Sex (male) | 1.14 | 1 | 1.07 |
| CCI | 1.55 | 1 | 1.24 |
| Insurance disease-type interaction | 1.96 | 5 | 1.07 |
| ICD-10 three-character group | 62.94 | 16 | 1.14 |
| Calendar quarter | 1.04 | 7 | 1.00 |
| Department-category interaction | 38.09 | 7 | 1.30 |

*GVIF = generalized variance inflation factor; df = degrees of freedom. A GVIF^(1/(2×df)) value < 2 indicates the absence of severe multicollinearity. CCI = Charlson Comorbidity Index.*

**Table S3. Hierarchical multivariable logistic regression for DIP high-magnification cases.** Patient-intrinsic model (Model 1) and full model (Model 2), with goodness-of-fit comparison.

| **Variable** | **OR** | **95% CI** | | **t** | | **P** |
| --- | --- | --- | --- | --- | --- | --- |
| (Intercept) | 0.009 | 0.007–0.011 | | -42.717 | | <0.001 |
| Age | 1.010 | 1.009–1.011 | | 18.813 | | <0.001 |
| Gender | 1.059 | 1.021–1.098 | | 3.090 | | 0.002 |
| CCI | 1.168 | 1.157–1.178 | | 34.141 | | <0.001 |
| Infectious & parasitic diseases | 5.861 | 4.532–7.580 | | 13.478 | | <0.001 |
| Neoplasms | 1.334 | 1.071–1.661 | | 2.569 | | 0.010 |
| Blood, haematopoietic & immune disorders | 8.593 | 6.730–10.970 | | 17.257 | | <0.001 |
| Endocrine, nutritional & metabolic diseases | 1.187 | 0.913–1.544 | | 1.281 | | 0.200 |
| Nervous system diseases | 2.852 | 2.222–3.661 | | 8.225 | | <0.001 |
| Circulatory system diseases | 1.649 | 1.318–2.061 | | 4.385 | | <0.001 |
| Respiratory system diseases | 3.848 | 3.045–4.862 | | 11.288 | | <0.001 |
| Digestive system diseases | 3.118 | 2.483–3.915 | | 9.793 | | <0.001 |
| Skin & subcutaneous tissue disorders | 6.925 | 5.338–8.984 | | 14.573 | | <0.001 |
| Musculoskeletal & connective tissue disorders | 1.869 | 1.469–2.379 | | 5.088 | | <0.001 |
| Genitourinary system diseases | 1.787 | 1.419–2.250 | | 4.939 | | <0.001 |
| Pregnancy, childbirth & puerperium | 6.885 | 5.241–9.045 | | 13.862 | | <0.001 |
| Congenital malformations & chromosomal anomalies | 2.438 | 1.799–3.304 | | 5.748 | | <0.001 |
| Symptoms, signs & abnormal findings, NEC | 3.294 | 2.486–4.364 | | 8.304 | | <0.001 |
| Injuries, poisoning & external cause sequelae | 4.970 | 3.902–6.329 | | 12.997 | | <0.001 |
| Health status & healthcare contact factors | 24.423 | 19.709–30.265 | | 29.203 | | <0.001 |
| **Goodness-of-fit metric** | **Model 1**  **(patient-intrinsic)** | | **Model 2**  **(full)** | | **Definition** | |
| Log-likelihood | -44271.182 | | -33350.695 | | Log-likelihood | |
| Null log-likelihood | -58589.270 | | -58507.285 | | Null log-likelihood | |
| Likelihood-ratio χ² (G2) | 28636.177 | | 50313.179 | | Likelihood-ratio chi-square | |
| McFadden pseudo-R² | 0.244 | | 0.430 | | McFadden pseudo-R² | |
| Cox–Snell R² | 0.145 | | 0.242 | | Cox–Snell R² | |
| Nagelkerke R² | 0.307 | | 0.509 | | Nagelkerke R² | |

*Model 1 included only demographic and clinical covariates (age, sex, diagnosis group, CCI). Model 2 (full model) additionally included cost-composition and length-of-stay variables. OR = odds ratio; CI = confidence interval; CCI = Charlson Comorbidity Index, DIP, Diagnosis-Intervention Packet. All listed coefficients were statistically significant (P < 0.001) except Endocrine, nutritional & metabolic diseases (P = 0.200). χ² = chi-square.*

**Table S4.** **Comparison of Charlson Comorbidity Index between high-magnification and non-high- magnification cases in the Secondary comprehensive category disease group**

| Characteristic | Overall  (N = 5,428) | Non-high- magnification  (N = 5,074) | High- magnification  (N = 354) | P value |
| --- | --- | --- | --- | --- |
| CCI, mean ± SD | 1.17 ± 1.67 | 1.13 ± 1.62 | 1.77 ± 2.22 | < 0.001 |
| CCI, median (MAD) | 0 (0.00) | 0 (0.00) | 1.00 (1.48) | — |
| CCI, trimmed mean ± SE | 0.84 ± 0.02 | 0.81 ± 0.02 | 1.39 ± 0.12 | — |

*CCI, Charlson Comorbidity Index; SD, standard deviation; MAD, median absolute deviation; SE, standard error; DIP, Diagnosis-Intervention Packet.P value was calculated using Welch’s two-sample t-test (t = −5.35, df = 379.8, p = 1.54 × 10−7). Mean difference (95% CI): −0.64 (−0.88, −0.41), indicating significantly higher CCI in the high-rate group. Median (MAD) and trimmed mean were not compared statistically; the t-test was performed on raw CCI values between groups.*

**Table S5**. **Quarterly trend of the proportion of high comorbidity burden cases (CCI ≥ 3) among all inpatients**

| **Quarter** | **CCI < 3** **n (%)** | **CCI ≥ 3** **n (%)** | **Total** **N** | **CCI ≥ 3** **Proportion (%)** |
| --- | --- | --- | --- | --- |
| 2024 Q1 | 16,674 (74.30) | 5,766 (25.70) | 22,440 | 25.70 |
| 2024 Q2 | 16,914 (72.34) | 6,467 (27.66) | 23,381 | 27.66 |
| 2024 Q3 | 17,950 (72.38) | 6,849 (27.62) | 24,799 | 27.62 |
| 2024 Q4 | 15,999 (70.23) | 6,782 (29.77) | 22,781 | 29.77 |
| 2025 Q1 | 15,756 (69.80) | 6,816 (30.20) | 22,572 | 30.20 |
| 2025 Q2 | 17,030 (69.66) | 7,419 (30.34) | 24,449 | 30.34 |
| 2025 Q3 | 19,417 (70.88) | 7,977 (29.12) | 27,394 | 29.12 |
| 2025 Q4 | 16,362 (69.11) | 7,312 (30.89) | 23,674 | 30.89 |
| **Overall** | **136,102 (71.08)** | **55,388 (28.92)** | **191,490** | **28.92** |

*CCI, Charlson Comorbidity Index. Data are presented as n (%) for each category. The proportion of CCI ≥ 3 cases increased from 25.70% in 2024 Q1 to 30.89% in 2025 Q4. Trend test: Cochran-Armitage test for trend, Z = −12.78, p < 2.2 × 10⁻¹⁶, indicating a statistically significant increasing trend in the proportion of high comorbidity burden cases (CCI ≥ 3) over the study period. This analysis included all hospital inpatients per quarter (not limited to the secondary comprehensive DIP subgroup) to assess the hospital-wide secular trend in case complexity.*
